# Contribution of Epithelial-Mesenchymal Transition to Pancreatic Cancer Progression

**DOI:** 10.3390/cancers2042084

**Published:** 2010-12-09

**Authors:** Seth B. Krantz, Mario A. Shields, Surabhi Dangi-Garimella, David J. Bentrem, Hidayatullah G. Munshi

**Affiliations:** 1 Departments of Surgery, Northwestern University Feinberg School of Medicine, Chicago, IL 60611, USA; E-Mail: dbentrem@nmff.org (D.J.B.); 2 Departments of Medicine, Northwestern University Feinberg School of Medicine, Chicago, IL 60611, USA; E-Mail: marioshields2012@u.northwestern.edu (M.A.S.); s-dangi-garimella@northwestern.edu (S.D.); 3 Robert H. Lurie Comprehensive Cancer Center, Northwestern University Feinberg School of Medicine, Chicago, IL 60611, USA; 4 Jesse Brown VA Medical Center, Northwestern University Feinberg School of Medicine, Chicago, IL 60612, USA

**Keywords:** pancreatic cancer, epithelial-mesenchymal transition microRNA, stem cells drug resistance

## Abstract

Pancreatic adenocarcinoma (PDAC) is one of the most lethal human malignancies, with median survival of less than one year and overall five-year survival of less than 5%. There is increasing evidence demonstrating that epithelial-mesenchymal transition (EMT) contributes to pancreatic cancer metastasis and to treatment resistance. In this review, we will examine the data demonstrating the role and regulation of EMT in pancreatic cancer progression, focusing particularly on the transcription factors and microRNAs involved in EMT. We will examine how EMT is involved in the generation and maintenance of stem cells, and the role of EMT in modulating resistance of PDAC cells to drug therapies. We will also identify putative EMT-targeting agents that may help to reduce the morbidity and mortality associated with pancreatic cancer.

## 1. Introduction

Pancreatic adenocarcinoma (PDAC) is one of the most lethal human malignancies and remains a daunting challenge for patients, clinicians, and researchers alike. There are approximately 43,000 new cases each year in the United States, with over 36,000 deaths, making it the fourth leading cause of cancer death [1]. Median survival is less than one year and overall five-year survival is less than 5% [2]. Additionally, over 80% of patients present with advanced disease not amenable to surgical resection, and even for those who do undergo surgery, treatment remains difficult with a five-year survival of only 20% [3,4,5]. Several factors are thought to contribute to the aggressive nature of pancreatic cancer. Anatomically, the location of the pancreas means patients are often asymptomatic until the disease is advanced, when they present with jaundice from obstruction of the bile ducts, or pain from invasion of the surrounding nerves. Histologically, PDAC is associated with a dense fibrotic reaction, known as the desmoplastic reaction, which is thought to contribute to disease progression and chemoresistance [6,7]. Despite improvements in surgical technique, enhanced imaging, and new chemotherapeutic agents, little progress has been made over the past 30 years in improving the survival of patients with PDAC [5,8]. Outcomes for patients remain extremely poor, and a better understanding of the cellular and biochemical factors that contribute to this terrible disease is essential if we are to make any significant improvements in the treatment of PDAC.

## 2. Epithelial to Mesenchymal Transition and Pancreatic Cancer

**Figure 1:**
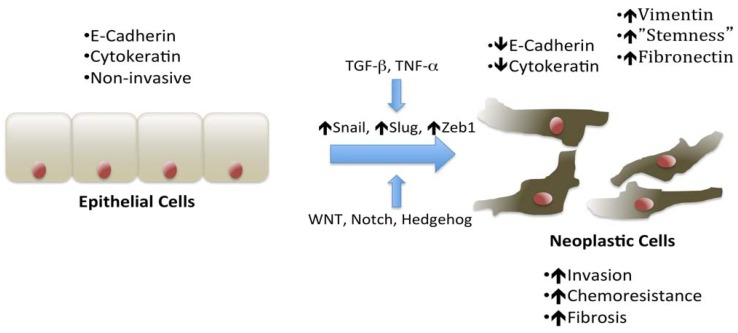
Schematic of epithelial to mesenchymal transition (EMT), including signaling pathways, transcription factors, and cell phenotype. Several cytokines and growth factors, notably TGF-β and TNF-α, along with the WNT, Notch, and Hedgehog pathways, induce EMT. The primary transcription factors in pancreatic cancer are Snail and Zeb1. Cell that undergo EMT lose their epithelial markers, such as E-cadherin and cytokeratin, and sheet like architecture and take on a mesenchymal phenotype with increased vimentin, fibronectin, and N-Cadherin expression along with single cell, spindle-like morphology. These cells can contribute to desmoplasia, are invasive, have stem-cell-like properties, and show increased chemoresistance.

An essential process for epithelial cancer cells to invade their basement membranes and subsequently metastasize to distant sites is that of epithelial to mesenchymal transition (EMT) [9,10,11]. As cells undergo EMT, they lose their epithelial features including loss of their sheet-like architecture, loss of polarity, and down regulation of E-cadherin; they also develop a mesenchymal phenotype, taking on a spindle-like, fusiform morphology, become motile, and start expressing mesenchymal markers, e.g., N-cadherin, fibronectin, and vimentin [10,12] (Figure 1). In human pancreatic tumor samples, fibronectin and vimentin are increased in high-grade tumors and within poorly differentiated areas of low-grade tumors [13]. This increase is associated with a corresponding decrease in E-cadherin expression. Significantly, patients with high vimentin and fibronectin and low E-cadherin expression have worse survival than those patients whose tumors demonstrate less evidence of EMT [13]. In a study based on a rapid autopsy program for patients with pancreatic cancer, 75% of the primary tumors with mesenchymal features developed metastatic lesions to the liver and lung [14].

As a dynamic process, EMT was initially characterized *in vitro* with some controversy over the role of EMT *in vivo* [15]. While a wide range of studies from patients with a variety of cancers have provided evidence for EMT *in vivo* [13,14,16,17,18], it has been argued that this is somewhat correlative in nature, and that rather than seeing the result of a cellular transition, it may merely reflect a change in cell population, with apoptosis of epithelial cells and proliferative expansion of fibroblastic cells [15]. However, lineage tracing studies in separate *in vivo* transgenic mouse models clearly demonstrate a role for EMT in intestinal fibrosis and breast cancer [19,20]. While mesenchymal proliferation and epithelial apoptosis may also be occurring, these two studies provide strong evidence of EMT *in vivo*, and thus lend further credibility to studies that examine EMT in human specimens utilizing gene and protein expression techniques.

**Figure 2:**
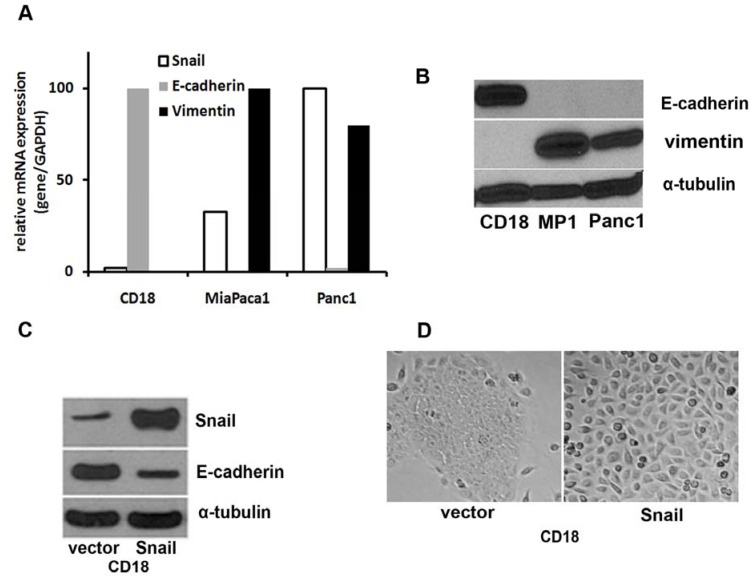
Snail expression is associated with decreased E-Cadherin expression, increased vimentin, and a fibroblast-like phenotype. (**A**) Snail, E-cadherin, and vimentin mRNA expression as determined by qRT-PCR, normalized to GAPDH, in three pancreatic cancer cell lines, HPAF-II/CD18 (CD18), MiaPaCa-1 (MP1), and Panc1. (**B**) E-cadherin expression inversely correlates with vimentin expression in three pancreatic cancer cell lines as determined by Western blot. (**C**) Expression of Snail and E-cadherin, as determined by Western blot, in CD-18 pancreatic cancer cells induced to express either Snail or a control vector. (**D**) Growth architecture of CD-18 pancreatic cancer cells induced to express either Snail or a control vector.

The key regulators of EMT include Snail, Slug, Zeb1, and Twist, which are zinc finger transcription factors that repress genes responsible for the epithelial phenotype (Figure 2) [9,10,11]. In resected PDAC specimens, nearly 80% have moderate to strong Snail expression while only 50% show similar Slug expression, with very few having strong Twist expression [16]. Snail expression is inversely correlated with E-cadherin expression, with decreased E-cadherin expression associated with higher tumor grade. Similar results were seen in pancreatic cell lines, with poorly differentiated lines showing higher levels of Snail and lower levels of E-cadherin compared with moderately differentiated cell lines [16]. Zeb1 expression in pathologic specimens also correlates with advanced tumor grade and worse outcomes [17,18,21]. In one study, tissue microarray analysis of pancreatic cancer showed an inverse relationship between Zeb1 and E-cadherin expression [18]. Furthermore, silencing of Zeb1 in pancreatic cancer cell lines led to the upregulation of E-cadherin and restoration of an epithelial phenotype [18]. Interestingly, Zeb1 was primarily responsible for the acquisition of an EMT phenotype, along with increased migration and invasion in response to NF-κB signaling in pancreatic cancer cells [21]. When reviewed in whole, the correlative *in vivo* data is recapitulated in the more experimental *in vitro* environment, lending strength to the role of EMT, specifically Snail and Zeb1 expression, in driving high grade tumors with decreased survival.

Transforming growth factor beta (TGF-β) is one of the primary drivers of EMT [7,10]. TGF-β can have both tumor suppressive and tumor promoting affects on pancreatic cancer [22,23,24]. Loss of Smad4 early in tumor development leads to loss of TGF-β growth inhibition and unchecked tumor growth in mouse models of pancreatic cancer. These tumors, however, are generally well differentiated [25,26]. Tumors with intact Smad4 signaling, meanwhile, are associated with an increase in EMT and subsequently are poorly differentiated [26]. Furthermore, these advanced tumors that have undergone EMT show increased tumor proliferation and migration in response to TGF-β [26]. Additionally, TGF-β-induced EMT may directly affect the growth pathways of various cancers, including pancreatic cancer. Over 90% of human pancreatic cancers have an activating mutation for the K-ras oncogene [27], on which the cancer is dependent for growth. Induction of EMT by TFG-β causes cells previously dependent on K-ras for continued growth to become K-ras independent. For example, shRNA ablation of mutant K-ras in the epithelial lung cancer cell line H358 led to an increase in cleaved caspase 3 along with decreased phosphorylated-AKT, while similar K-ras ablation in H358 cells that had undergone EMT following TGF-β treatment showed no increase in cleaved caspase or decrease in phosphorylated-AKT [28]. Conversely, K-ras independent cells forced to undergo mesenchymal to epithelial transformation (MET) by targeting Zeb1 with shRNA subsequently become K-ras dependent [28]. Similar K-ras dependency on EMT was seen in pancreatic cancer cells [28]. Thus, as cells undergo EMT, tumors that once may have responded to interruption of oncogenic signaling pathways can become unresponsive [28]. With the increase in targeted therapies, many of which are specifically designed to target growth pathways, EMT may have additional implications in drug resistance and cancer progression.

Inflammation plays a significant role in pancreatic cancer [29,30], and inflammatory signaling through NF-κB has been shown to increase both EMT and cancer cell invasion. Snail activity is increased via stabilization at the protein level in response to TNF-α driven NF-κB signaling [31]. Additionally, knockdown of Snail in this system abrogated TNF-α driven cancer cell migration and invasion [31]. A similar interaction between TNF-α, NF-κB and EMT was demonstrated in pancreatic cancer cells, with transfection with a dominant negative form of IκBα abrogating TNF-α-induced EMT [21]. Interestingly, these authors also found evidence of interaction between NF-κB and TGF-β pathways in the induction of EMT in pancreatic cancer. TGF-β-induced EMT was completely blocked in pancreatic cells expressing the dominant negative form of IκBα, suggesting that TGF-β induced EMT is dependent on intact NF-κB signaling [21]. In addition to being necessary for TGF-β induced EMT, NF-κB could also promote an EMT phenotype independent of Smad signaling. In Smad4 null IMIM PC-2 pancreatic cancer cells, expression of the dominant active IKK2 led to EMT through increased MAPK/ERK signaling [21].

## 3. Role of microRNAs in Modulating EMT in Pancreatic Cancer

MicroRNAs, small single-stranded non-coding RNAs, have been reported in many cancers, including pancreatic cancer [32,33,34,35]. As they do not code for any protein, microRNAs exert their effects by interacting directly with mRNA, often serving to either degrade or prevent translation of the message [35]. They play a role in numerous cell and developmental processes. Most relevant to cancer, they affect differentiation, proliferation, and apoptosis and can serve as either tumor promoters or suppressors depending on their downstream effects [32,33,34,35]. MicroRNAs of the miR-200 family (miR-200a, b, c, miR-141 and miR-429) and miR-205 have been identified as key negative regulators of both EMT and the metastatic ability of cancer cells [36,37]. These microRNAs are downregulated in high grade and poorly differentiated tumors, while forced expression of miR-200 microRNAs has been shown to inhibit TGF-β1-induced EMT in MDCK cells. In lung cells, forced miR-200 expression abrogated the cells’ invasive and metastatic abilities. The miR-200 family targets the key regulators of EMT, such Zeb1 and Sip1 (also known as Zeb2), and as such leads to increased E-cadherin levels [36,37].

Recent surveys of global microRNA expression patterns in pancreatic cancer cell lines showed that 39 microRNAs, including the miR-200 family, were deregulated and have at least two-fold differential expression in PDAC cell lines compared to control nontransformed pancreatic ductal cell lines [38]. Expression of miR-200 family members correlated positively with E-cadherin expression and negatively with the miR-200 target Zeb1 [38]. High levels of miR-200c expression strongly correlated with E-cadherin levels in resected human pancreatic tumor samples and were associated with significantly better survival rates compared with patients whose tumors had low levels of miR-200c expression [39]. Interestingly, Zeb1 can also directly suppress transcription of miR-200 family members miR-141 and miR-200c [40] indicating an interplay between Zeb1 and miR-200 family that regulates the differentiation state of pancreatic cancer cells.

## 4. Contribution of EMT to Stem Cells in Pancreatic Cancer

There is increasing interest in the subpopulation of cells within tumors that have stem cell-like properties [41,42,43]. These cells are frequently associated with metastatic foci and chemoresistance and are increasingly linked to an EMT phenotype [44,45,46]. Both TGF-β and Snail expression have been shown to increase the stemness of immortalized mammary cells. These cells show an increased ability to form mammospheres in 3D cultures, develop ductal outgrowths in xenotransplant assays, and have an increased population of CD44-high/CD24-low cells, an expression profile associated with stem-cell like behavior [42,46]. Further, CD44-high/CD24-low cells isolated from primary breast tumors show increased expression of EMT markers and EMT-inducing transcription factors [46]. These cells are also associated with chemoresistance, as cells found in residual tumors following standard chemotherapy demonstrate increased levels of EMT markers and stain CD44-high/CD24-low [44,46].

**Figure 3:**
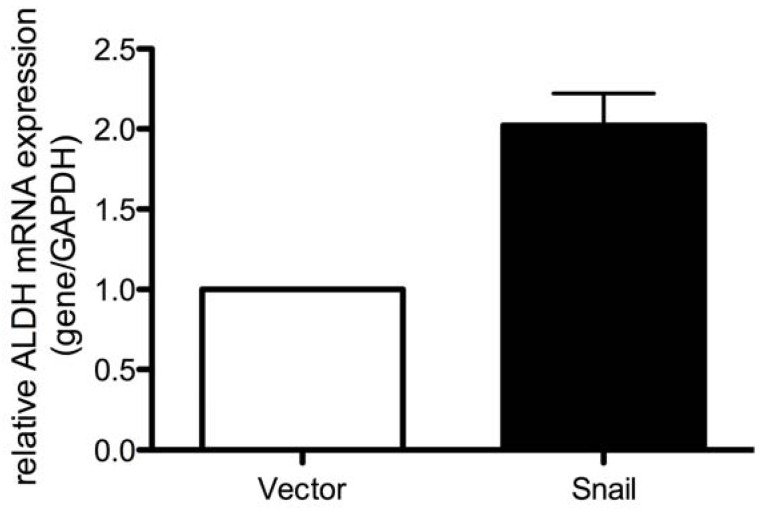
Expression of the stem cell marker aldehyde dehydrogenase is associated with increased expression of Snail. Graph showing a comparison of aldehyde dehydrogenase (ALDH) expression, as measured by qRT-PCR, normalized to GAPDH, in CD-18 pancreatic cancer cells induced to express either Snail or a control vector.

There is also evidence to suggest the existence of cancer stem cells in pancreatic cancer [47,48]. These cells are CD44-high/CD24-high and express epithelial specific antigen (ESA) [47]. Although the CD44-high/CD24-high/ESA-high cells comprise a small population of any particular pancreatic tumor, these cells have the ability to self-renew and reproduce the original tumor heterogeneity. Recently, using aldehyde dehydrogenase (ALDH) activity as a more specific marker of cancer stem cells, it was shown that ALDH-high cells comprise an even more select subpopulation of cells in human pancreatic cancers that are tumorigenic and capable of producing tumors at very low numbers [14]. These ALDH-high cells have reduced E-cadherin expression and increased Slug expression [14]. Interestingly, overexpression of Snail in pancreatic cancer cells leads to increased ALDH expression (Figure 3). ALDH-high cells with a mesenchymal phenotype have also been found in metastatic lesions of patients with pancreatic cancer [14]. Further, microRNAs that are associated with EMT, such as the miR-200 family, which are repressed by Zeb1 [49], also regulate stem cell behavior [49,50]. miR-200c cooperates with other microRNAs to suppress expression of stem cell factors, such as Bmi1, Sox2 and KLF4 in cancer cells and mouse embryonic stem cells [49,50]. Expression of miR-200c in breast cancer stem cells prevented the formation of tumors *in vivo* and even prevented new duct formation by non-malignant mammary stem cells [50].

## 5. Importance of EMT in Enhancing Drug Resistance in Pancreatic Cancer

Pancreatic cancer remains extremely lethal in large part due to the poor efficacy of existing treatments [8,51]. EMT has been shown to be a significant contributor to chemo-resistance in several cancers and in pancreatic cancer in particular [18,52,53,54]. Induction of gemcitabine resistance in previously sensitive cell lines led to the development of cells with an EMT phenotype. The cells took on a more spindle shaped morphology, showed increased vimentin, and decreased expression of E-cadherin along with increased cytoplasmic localization. Further, the cells had an increased migratory and invasive ability compared to gemcitabine sensitive cells [54]. Gene expression profiling of chemoresistant cell lines showed a strong association between expression of genes associated with EMT and chemotherapy resistance [18]. The EMT transcription factor Zeb1 was upregulated in resistant cell lines and correlated with decreased expression of E-cadherin. Silencing of Zeb1 with siRNA led to mesenchymal to epithelial transition (MET) with restoration of chemosensitivity [18]. This relationship was validated prospectively in five additional pancreatic cancer cell lines that were analyzed according to their Zeb1 levels. Lines with higher Zeb1 expression and an EMT phenotype showed increased drug resistance [18]. Maintenance of chemoresistance in cell lines that have undergone EMT is dependent on Notch and NF-κB signaling [53]. Inhibition of Notch-2 led to down regulation of Zeb1, Snail, Slug, and NF-κB, along with attenuation of the migratory and invasive capacity of the gemcitabine resistant cells. The role of NF-κB in promoting EMT and gemcitabine is further evidence linking EMT, inflammatory signaling pathways and chemo-resistance [53].

EMT may play a role in modulating resistance not only to traditional chemotherapies, but to targeted biologic therapies as well. Cells that expressed either mutated E-cadherin, or had high levels of Snail, Zeb1, and vimentin, and thus a mesenchymal phenotype, showed significantly decreased growth inhibition in response to erlotinib treatment than cells with an epithelial phenotype [17]. Further, cells from the same patient showed differential response to drug treatment, with cells from the primary tumor being responsive while cells harvested from a liver metastases, and which demonstrated a mesenchymal phenotype, were resistant to erlotinib [17].

Pancreatic cancer cells may not be inherently chemoresistant [54]. The pronounced fibrotic reaction, primarily generated by myofibroblast-like stellate cells [55,56,57], may limit the delivery of current chemotherapeutic agents. While quiescent fibroblasts within the microenvironment are activated by TGF-β [58], a significant number of myofibroblasts have in fact been shown to arise from epithelial cells that have undergone EMT [59]. In the adult kidney, activation of Snail is sufficient to cause renal fibrosis [60], while Hedgehog signaling, which has been shown to contribute to EMT [61], was recently shown in pancreatic cancer to contribute to resistance to gemcitabine through modulation of the tumor microenvironment, specifically by affecting the stroma and type I collagen [6,14,53]. Thus, EMT may modulate chemoresistance not only within cancer cells themselves, but also by modulating the tumor microenvironment by promoting a desmoplastic reaction.

## 6. Targeting EMT to Reduce the Morbidity and Mortality of Pancreatic Cancer

Given the role of EMT in promoting chemoresistance, invasion, and stem cell-like properties, specifically targeting EMT could potentially serve to decrease metastasis and overcome drug resistance, though significant additional work is needed to translate these findings into meaningful therapies. Restoring expression of miR-200 family micro-RNAs or, alternatively, targeting EMT signaling pathways such as Notch and Hedgehog, or their ultimate downstream mediators the EMT-inducing transcription factors, such as Zeb1, could also restore the epithelial state and make the tumors more sensitive to therapeutic agents. *In vitro* methods, such as direct delivery of microRNAs or utilizing siRNAs against transcription factors, have yet to be translated to the *in vivo* environment due to a number of technical barriers related to safety, delivery and efficacy [62,63]. For this reason, there is increasing interest in using compounds that can modulate EMT-inducing microRNAs or transcription factors. Recently, it was shown that treating pancreatic cancer cells with the circumin analogue CDF restored miR-200 levels and sensitized the pancreatic cancer cells to gemcitabine treatment *in vitro* [64]. In prostate cancer cells, Silibinin, a naturally occurring flavanoid, has been shown to attenuate EMT by down-regulating expression of Zeb1 and Slug [65]. Interestingly, the anti-diabetic drug metformin may play a role in cancer therapy by decreasing stem cell-like populations through modulation of EMT pathways [66]. Treatment of breast cancer cells with metformin led to a reduced CD44-high/CD24-low population, decreasing the ability of breast cancer stem cells to form mammospheres. Additionally, there was decreased expression of the key drivers of EMT, including Zeb1, Twist1 and Slug, with lower TGF-β levels.

Given the essential role of TGF-β in EMT, it has also gained attention as a therapeutic target. Neutralizing antibodies against TGF-β are currently in Phase I clinical trials for renal cancer and malignant melanoma (http://clinicaltrials.gov/ct2/show/NCT00356460). In addition, a peptide that blocks binding of TGF-β1 to its receptor is in a Phase II clinical trial to attenuate skin fibrosis in patients with systemic sclerosis (http://clinicaltrials.gov/ct2/show/NCT00574613).

Clinical trials targeting Hedgehog and Notch signaling—known EMT pathways that have been implicated in cancer stem cells and chemoresistance [6,14,53]—are also underway. A current phase II trial is looking at the Hedgehog inhibitor GDC-0449 as combination therapy in patients with metastatic disease (http://clinicaltrials.gov/ct2/show/NCT01088815) while a separate pilot study is looking at GDC-0449 with a specific focus on its ability to target cancer stem cells (http://clinicaltrials.gov/ct2/show/NCT01195415). Additionally, there are two phase I trials of Notch inhibitors (http://clinicaltrials.gov/ct2/show/NCT01098344 and http://clinicaltrials.gov/ct2/show/NCT01145456) that are currently recruiting.

The relationship between EMT and cancer stem cells was recently highlighted by a screening study looking for compounds that specifically target cells that have undergone EMT. Compounds that were effective against cells that had undergone EMT were also cytotoxic against cancer stem cells. One compound, salinomycin, decreased the number of mammospheres formed by breast cancer cells *in vitro*, while inhibiting tumor growth and promoting differentiation *in vivo* [41]. Importantly, this screen demonstrated that specifically targeting cells that have undergone EMT also targets cancer stem cells, which has important implications for treating patients with metastatic disease and disease refractory to standard chemotherapy. While all of these therapies hold great promise, they remain to be validated in clinical trials, and until the results of ongoing and future trials are known, they remain speculative, however promising.

## 7. Conclusions

Targeting EMT holds significant promise in treating a range of malignancies and in pancreatic cancer specifically. Given the role of EMT in multiple aspects of cancer progression, targeting EMT could contribute both to increased sensitivity to standard chemotherapy while also improving response towards growth factor directed therapies, such as those against EGFR signaling. Targeting EMT may also attenuate fibrosis, thereby increasing delivery of drugs to cancer cells, and help to reduce the population of cancer stem cells that are thought to contribute to metastatic disease and treatment resistance. As of today, this remains extremely promising, but ultimately the benefits are unknown. We hope to gain more insight into the efficacy of targeting these pathways from the ongoing clinical trials. Still, as we further understand the role of EMT in pancreatic cancer progression and identify additional regulators of EMT, this will hopefully increase the number of drugs targeting EMT and may in turn help to reduce the morbidity and mortality associated with pancreatic cancer.
